# Gender-Specific Prevalence of Risk Factors for Non-Communicable Diseases by Health Service Use among Schoolteachers in Afghanistan

**DOI:** 10.3390/ijerph18115729

**Published:** 2021-05-26

**Authors:** Sharifullah Alemi, Keiko Nakamura, Ahmad Shekib Arab, Mohammad Omar Mashal, Yuri Tashiro, Kaoruko Seino, Shafiqullah Hemat

**Affiliations:** 1 Department of Global Health Entrepreneurship, Division of Public Health, Tokyo Medical and Dental University, Tokyo 113-8519, Japan; alemi.ith@tmd.ac.jp (S.A.); dr.arab03@yahoo.com (A.S.A.); ytashi.ith@tmd.ac.jp (Y.T.); seino.ith@tmd.ac.jp (K.S.); 2 French Medical Institute for Mothers and Children (FMIC), Kabul 1011, Afghanistan; omer_mashal@yahoo.com; 3 Ministry of Public Health, Kabul 1002, Afghanistan; shafiqullahhemat8@gmail.com

**Keywords:** biomedical indicators, non-communicable diseases, female teachers, lifestyle behaviors

## Abstract

Objectives of this study were: (1) to examine gender differences in biomedical indicators, lifestyle behaviors, self-health check practices, receipt of professional non-communicable disease (NCD)-related lifestyle advice, and the use of health services among teachers in Afghanistan; and (2) to seek the patterns of these indicators among users and non-users of health services among both male and female teachers. This cross-sectional study was carried out among 600 schoolteachers in Kabul city in February 2017. Gender differences in percentage distributions of abnormal biomedical indicators, lifestyle behaviors, self-health check practices, and receipt of professional lifestyle advice were examined. These patterns were further analyzed according to the use of health services in the previous 12 months by both genders. The results showed that male teachers had a higher prevalence of hypertension, increased serum triglycerides, physically active lifestyle, and tobacco use than female teachers (28.2/20.4, *p* = 0.038; 47.0/37.9, *p* = 0.040; 54.3/40.9, *p* = 0.002; 15.8/0.7, *p* < 0.001, respectively); female teachers had a higher prevalence of increased serum LDL cholesterol, overweight/obesity, and frequent consumption of fruits/vegetables than male teachers (61.3/50.8, *p* = 0.018; 64.7/43.5, *p* < 0.001; 71.4/53.8, *p* < 0.001, respectively). Female teachers were more likely to receive professional lifestyle advice related to NCDs than male teachers. Although users of health services practiced self-health checks and received professional lifestyle advice more frequently than non-users, abnormal biomedical indicators were similarly shown among users and non-users of health services in both genders. In conclusion, high prevalence of abnormal biomedical indicators was indicated in both male and female teachers, although the specific abnormal biomedical indicators differed by gender. Users and non-users of health services presented a similar prevalence of these abnormal indicators. Understanding the differences in patterns of NCD risk factors is essential when developing gender-informed policies.

## 1. Introduction

Non-communicable diseases (NCDs) are the leading cause of death worldwide, particularly in low- and middle-income countries, wherein nearly three-quarters of all NCD deaths occur [1]. NCDs arise from multiple overlapping behavioral and clinical risk factors that act singly or in combination [2]. Prevention and control of NCDs would primarily involve controlling and reversing the rise of related risk factors in the target population [3,4]. Healthy lifestyle interventions and initiatives should be at the forefront of strategic directions within the health system to effectively combat NCDs [5].

The development and course of NCD risk factors differ when viewed through the lens of gender [6]. According to the 2019 Global Burden of Disease Study, the proportion of attributable deaths due to leading risk factors differed in men and women. The top risk for attributable deaths among women was high systolic blood pressure (SBP), followed by dietary risks, elevated fasting plasma glucose (FPG), and high body mass index (BMI), contributing to 20.3%, 13.5%, 11.9%, and 9.8% of all female deaths in 2019, respectively. For men, the leading risks were tobacco use, high SBP, dietary risks, and high FPG, accounting for 21.4%, 18.2%, 14.6%, and 11.1% of all male deaths in 2019, respectively [7]. NCDs are often referred to as lifestyle-related diseases, and their risk factors are largely preventable through early detection and improvements in lifestyle behaviors [3,8].

In recent decades, the disease spectrum has shifted from communicable diseases to NCDs in Afghanistan, imposing a double burden of diseases, with NCDs estimated as responsible for 44% of all deaths, males accounting for 51% and females for 49% of all premature deaths from NCDs [1,9]. The share of NCDs in disability-adjusted life-years (DALYs) increased from 28.8% to 42.6% between 1990 and 2017 [9]. In a low-resource country like Afghanistan, developing and implementing supportive policies is crucial to reducing exposure to risk factors and decreasing avoidable morbidity and mortality from NCDs [10]. NCDs are currently a policy priority in the Afghanistan health sector. The National Health Strategy 2016–2020 stresses increasing awareness and advocacy and strengthening human and institutional capacity to prevent and control NCDs [11].

Schoolteachers form one of the largest workforces in the world. They are key to shaping children’s behaviors and habits. Teachers’ health is reported to significantly impact their teaching quality and the learning success of their students [12]. Studies on occupational health focus on research into and prevention of occupational diseases, as certain work practices harm employees’ health. Similarly, identifying workplace risks for teachers and overcoming these for higher productivity is of great importance. Risk factors in the workplace can cause or accelerate NCDs [13]. Employees with unhealthy habits have high rates of lost productivity [14]. Therefore, teachers must maintain healthy lifestyle behaviors, including eating balanced diets, and engaging in habitual physical exercise. Improving teachers’ health can boost their productivity and reduce absenteeism.

In Afghanistan, healthcare facilities offer various diagnostic and therapeutic services for NCDs, and some of the facilities have guidelines, trained staff, medications, and medical supplies related to NCDs [15]. Regular health checks may allow early detection of problems when they are easier to treat [16]. A meta-analysis of six trials on practice-based health checks and their effects demonstrated that the BMI, blood pressure, and total cholesterol level improved significantly in the regular health-check group and that the number of patients at high risk for NCDs also decreased [17]. The prevalence of adverse lipid and diabetic profiles and elevated blood pressure levels has been well studied among food-secure and food-insecure schoolteachers in Kabul [18]. It is necessary to understand gender differences in the risk of NCDs according to the status of health service use, which would help provide tailored interventions to accelerate the prevention and control of NCDs. In this study, we analyzed gender differences in the prevalence of hypertension, increased glycosylated hemoglobin (HbA1c), and abnormal lipid profiles, as well as the behavioral indicators of NCDs among schoolteachers, according to the status of health service use by schoolteachers in the previous 12 months. Such information will be useful for advocacy of, and decision-making on, prevention and management of NCDs across multiple healthcare system levels.

## 2. Methods

### 2.1. Study Design and Sample

This cross-sectional study was carried out in 2017 among 600 currently working male and female teachers of primary, secondary, and high schools in Kabul, which were collaboratively selected by the joint committee of the Ministry of Public Health (MoPH) and Ministry of Education (MoE), Afghanistan. The male-to-female teacher ratio was not predetermined and was based on voluntary participation. The participants were selected from all 22 municipal districts of Kabul, comprised of residents from different provinces and ethnicities. The inclusion criteria were as follows: (1) permanent schoolteachers (both male and female) and (2) schoolteachers committed to volunteer work as a school health educator. Trainees and short-term contracted schoolteachers were excluded. For sample selection, all 270 public schools in Kabul were contacted, but teachers from 210 schools voluntarily attended. The study sample was randomly selected from a list of eligible participants. The sample size calculation is discussed in detail in an earlier study [18].

### 2.2. School and Teacher Involvement

The study team worked with the joint committee of the MoPH and MoE, Afghanistan. School principals were invited to a program of health promotion at schools in Afghanistan for orientation and assisting in preparing the lists of eligible schoolteachers. According to the consultations with schools, needs relevant to the assessment of health and lifestyle behaviors of schoolteachers and training of schoolteachers in order to gain knowledge and skills in the prevention of NCDs were identified. Schools were involved in the conduct of the study by facilitating recruitment of participants and coordinating health examinations. Schools were involved in a plan of dissemination of the study results to participating teachers, who were later invited to meetings to report the study results and to training on prevention of NCDs.

### 2.3. Study Variables

#### Stratification Variables

The stratification variables used for the analysis were gender (male/female) and status of use of health services (users/non-users). A history of at least one visit to a health facility (health center, hospital, or private clinic) in the previous 12 months was regarded as an indicator of health service use.

### 2.4. Study Tools and Measurement Procedure

Data were collected through questionnaire interviews, anthropometric measurements, and blood biochemistry tests. The survey instrument was adapted from a reference questionnaire developed by a joint research team from the Tokyo Medical and Dental University and contained items for a detailed evaluation of the participants’ sociodemographic profiles, general health status, dietary habits, physical activity levels, and tobacco use status. The questionnaire was translated from English into one of Afghanistan’s national languages (Dari) and was adjusted considering Afghanistan’s specific context. Anthropometric measurements included height and weight as well as blood pressure using standardized instruments and protocols. Height was measured using a free-standing stadiometer and recorded in centimeters (cm). Body weight was measured using a pre-calibrated weighing scale, when the participants were barefoot and wore light clothes, and was recorded in kilograms (kg). BMI was calculated as body weight (in kg) divided by height (in meters (m)) squared.

After the participants had rested for at least 5 min, trained health workers measured their blood pressure in a sitting position with the arm supported at the heart level. The blood pressure was measured twice with a 3- to 5-min interval between the two measurements, using an automated blood pressure cuff of appropriate size (OMRON, Kyoto, Japan). The average of the two measurements was used for the analysis. Next, blood biochemistry tests were performed, including measurement of HbA1c, total cholesterol, low-density lipoprotein (LDL) cholesterol, high-density lipoprotein (HDL) cholesterol, and triglycerides. Certified phlebotomists collected blood samples from each participant after obtaining their written consent for the laboratory examinations. HbA1c was measured using fully automated boronate affinity chromatography (Clover A1c) with an estimated time of 5 min, and the lipid profile was measured using a kit on a semi-automated clinical chemistry analyzer (Micro-lab 300, Komax Group, Dierikon, Switzerland). All blood tests were performed at a laboratory in Kabul.

#### 2.4.1. Sociodemographic Variables

Self-reported sociodemographic variables included age group, marital status, education level, teaching experience, and perception of general health status.

#### 2.4.2. Biomedical Indicators of NCDs and BMI

The six biomedical indicators of NCDs examined were blood pressure, HbA1c, total cholesterol, LDL cholesterol, HDL cholesterol, and triglycerides. Abnormal levels of the biomedical indicators were high blood pressure (≥130/85 mmHg), high HbA1c (≥5.5%), high cholesterol (≥200 mg/dL), high LDL cholesterol (≥100 mg/dL), low HDL cholesterol (<40 mg/dL), and high triglyceride levels (≥150 mg/dL). Based on internationally recognized BMI values for adults, values ≥ 25.0 kg/m^2^ were considered in the overweight/obese range. The presence of multiple unfavorable conditions included three or more comorbidities. The high blood pressure levels were set according to the 2017 American College of Cardiology/American Heart Association guidelines for the prevention, detection, evaluation, and management of high blood pressure in adults [19]. The cut-off value for HbA1c was determined based on a study investigating the threshold of HbA1c for screening of diabetes [20] and a systematic review of HbA1c level and risk of diabetes [21]. LDL, Total, and HDL cholesterol cut-off values were decided based on Adult Treatment Panel III (ATP-III) guidelines [22] and previous studies [23,24].

#### 2.4.3. Lifestyle Behavior Indicators of NCDs

Questionnaire on lifestyle behaviors included questions about consumption of food items, physical activity, and tobacco use. Consumption of different food items was measured using the following question, ‘How many times did you consume the following food items in the previous seven days?’ The food items included fruits; raw and cooked vegetables; soft drinks; cakes; fried, processed or fast foods; red meat; chicken; beans; potatoes; sweets; rice; bread and nuts. The responses were recorded as: never; 1–3 days per week; 4–6 days per week; or every day. For this study, consumption of fruits and vegetables was used, and the response options were categorized into <4 times per week or ≥4 times per week. Physical activity was measured using the following question, ‘On average, how long did you engage in the following activities per day?’. The response options included physical exercise, walking, and other daily activities, with the length of each activity ranging from: not at all, 10–30 min, 30–60 min, 1–2 h, 2–3 h, or more than 3 h. Information on physical exercise and walking was used in the study to assess physical activity. The response options to physical exercise/walking were coded into categories: ‘<1 h per day’ or ‘≥1 h per day’. Tobacco use was measured using the following question, ‘Do you currently use any of the following tobacco products?’. The response options included cigarettes, snuff (smokeless tobacco), or water pipes. The responses were categorized into ‘yes’ if participants currently used any tobacco products and ‘no’.

#### 2.4.4. Self-Health Check Practices

Self-health checks referred to measurement of blood pressure, blood glucose, and cholesterol in the previous 12 months.

#### 2.4.5. Health Professional Advice Variables

Participants were asked, “During the previous 12 months, has any doctor or other health professional offered you advice to consume at least five servings of fruits/vegetables each day, reduce your dietary fat intake, reduce your dietary salt intake, engage in habitual physical exercise or increase your physical activity levels, lose/maintain weight, or avoid tobacco use?” The response options were dichotomized into no (not received lifestyle modification advice) or yes (received lifestyle modification advice), and the responses were self-reported. Participants who were on medication for hypertension, diabetes, and hyperlipidemia were excluded from the analysis of biomedical indicators related to NCDs in order to avoid potential bias.

### 2.5. Statistical Methods and Analysis Plans

All statistical analyses were performed using Stata version 15.1 (StataCorp, College Station, TX, USA). We used the chi-squared test to compare gender differences across the biomedical and lifestyle behavior indicators of NCDs, self-health check practices, and health professional advice variables according to the status of health service use in the previous 12 months. Age-specific prevalence of biomedical indicators was also calculated. *p* < 0.05 was considered indicative of statistical significance.

## 3. Results

Of the 600 teachers included in the analyses, 416 (69.3%) were female. The mean age of the participants was 41.9 years. As for the status of health service use, 60% of the participants reported having used health services (visit to health center, hospital, or private clinic) in the previous 12 months. Differences at *p* < 0.05 were observed for all variables between males and females, except for teaching experience and status of health service use. Details regarding other characteristics of the participants stratified by gender are presented in Table 1.

Table 2 shows the prevalence of biomedical indicators related to NCDs, comorbidities, and lifestyle behaviors by gender. Males showed a higher prevalence of hypertension and increased triglyceride levels than females; however, LDL cholesterol levels were more frequently elevated and BMI values were more frequently in the overweight/obese range in females. In terms of lifestyle behaviors, males led a more physically active lifestyle than females. The proportion of participants consuming fruits/vegetables frequently was higher among females, while tobacco use prevalence tended to be higher among males. The percentages of self-health checks (measurement of blood pressure, blood glucose, and cholesterol) and receipt of professional lifestyle advice related to NCDs (advice to reduce fat, salt, start physical exercise or increase physical activities, and lose/maintain body weight) were higher in females than in males.

Comparison of the mean values of biomedical indicators among male and female teachers by age group is detailed in Table 3. A trend towards higher mean systolic and diastolic blood pressures was observed as the age group increased among both males and females. The mean systolic and diastolic blood pressures were higher in teachers aged 51 and over than in other age groups. The mean HbA1c level also showed an increasing trend as the age group increased among males and females. The mean HbA1c level was the highest in the age group of 51 and over.

The distribution of total cholesterol, LDL cholesterol, HDL cholesterol, and triglyceride levels showed a different pattern depending on gender. Among males, the mean levels of total cholesterol increased with age, and the highest values were observed among teachers aged 51 and over. However, the mean levels of LDL cholesterol and triglycerides were the highest among teachers aged 41–50 years. In contrast, the mean HDL cholesterol level decreased as age increased, and the lowest mean value was recorded among teachers aged 51 and over. Among females, the mean total cholesterol, LDL cholesterol, and triglycerides levels were the highest among teachers aged 51 years or older. The lowest mean HDL cholesterol value was also recorded among the teachers aged 51 and older. The mean total cholesterol and LDL cholesterol levels were higher in females than in males across all age groups. In contrast, the mean triglyceride level was higher in males than in females across all age groups. Females had lower mean HDL cholesterol levels than males. The mean (SD) BMI scores of participants were 24.50 ± 3.86 and 27.01 ± 4.84 for males and females, respectively. Among both males and females, the mean BMI level was highest among teachers aged 41–50 years, followed by those aged 51 and over. In addition, compared to males, females showed a higher mean BMI value across all age groups.

Overall, the percentage of users of health services who had undergone measurement of their blood pressure, blood glucose, and cholesterol was significantly higher than that of non-users of health services. Furthermore, a relatively high percentage of users of health services had received advice to consume at least five servings of fruits/vegetables each day, reduce their fat and salt, and lose/maintain their body weight as compared to the percentage of non-users of health services. The proportion of participants who practiced habitual exercise or walking for more than 1 h per day was higher among non-users of health services than among users of health services (Table 4).

The prevalence of the biomedical indicators related to NCDs and comorbidities by gender and status of health service use in the previous 12 months is presented in Table 5. Among males, a significantly higher percentage of users of health services had increased LDL cholesterol than non-users of health services. For all other variables, the comparison between these two groups resulted in a difference that was not statistically significant. A gender-stratified prevalence of lifestyle behaviors by biomedical indicators related to NCDs is provided in the Supplementary Material, Table S1.

## 4. Discussion

This is the first study to examine gender differences in the patterns of biomedical and behavioral indicators of NCDs among schoolteachers in Afghanistan. This study documented significant gender differences in the prevalence of biomedical and behavioral indicators of NCDs, with differences varying according to the status of health service use. Overall, males showed a higher prevalence of hypertension, increased triglyceride levels, physically active lifestyle, and tobacco use than females. Furthermore, more females had significantly higher prevalence of BMI values in the overweight/obesity range, elevated LDL cholesterol, and more frequent consumption of fruits/vegetables than males. This underscores the need for improving access to and quality of health services to curtail the problem of NCDs.

### 4.1. Health Service Use

About 60% of all the teachers surveyed in this study had used routine health services in the previous 12 months. Most of those who had not used health services might be under the notion that they were in better health, and did not need to visit healthcare facilities. In contrast, the high prevalence of biomedical indicators suggests that the use of health services among teachers needs to improve. According to a recent survey in Afghanistan, the key barriers to the use of health services were inaccessibility to healthcare facilities, unhelpful personnel, inappropriate hours of services that conflicted with the working hours of the participants, lack of security, lack of female healthcare providers, and lack of male companions [25]. Any of these obstacles could also explain the poor use of health services by teachers. Regular health checks are essential for early detection and management of NCD risk factors. According to a study in India, several chronic clinical abnormalities were newly detected in apparently healthy individuals through a Master Health Checkup [26]. More frequent visits to healthcare facilities were linked with reasonable control of the lipid profile and blood pressure [27]. In addition, school health checkup programs may help identify schoolteachers and students with or at risk of NCDs.

### 4.2. Prevalence of Biomedical Indicators of NCDs and BMI

In this study, overall, teachers showed a high prevalence of biomedical indicators of NCDs. A pilot study on the risk factors for cardiovascular disease conducted among teachers in South Africa also demonstrated higher prevalence of risk factors, including hypertension, hypercholesterolemia, diabetes, and BMI values in the overweight/obesity range (48.5%, 20.5%, 10.1%, and 84.7%, respectively) [28]. Some common cardiovascular risk factors were reported to occur with high prevalence among Nigerian teachers, including hypertension (36.1%), increased total cholesterol (38.6%), decreased HDL cholesterol (37.3%), and increased LDL cholesterol (56.6%) [29]. Primary healthcare efforts are needed to reverse the rising trend of NCDs among teachers in Afghanistan. Regular monitoring of the NCD biomarkers could lead to early diagnosis and effective treatment. NCD management is complex and requires prompt consideration from decision-makers in health policy and healthcare systems [30]. It also calls for multi-sectoral and multi-level approaches among individuals, healthcare providers, community members, and policymakers [13].

### 4.3. Lifestyle Behaviors

Unhealthy lifestyle behaviors are recognized causes of NCDs. Male and female teachers are exposed to varying behavioral risk factors for NCDs. In this study, female teachers were found to be less physically active than their male counterparts. Studies conducted in other countries have also reported lower physical activity levels among women than men [31,32,33]. Traditional norms, cultural values, and/or lack of support in the community may contribute to women’s poorer engagement in physical activities [32]. The barriers to Afghan women participating in physical exercise, particularly regular exercise, are numerous and complex. The Afghan family’s patriarchal and conservative social structure has influenced women to stay at home and avoid physical activities outside the house. Insufficient physical activity could also be due to insufficient female-friendly outdoor spaces for physical exercise and a lack of women-only gyms in both urban and rural areas. The low prevalence of sufficient physical activity among female teachers might also be due to their lower awareness of physical exercise benefits and the lack of sufficient time away from heavy school workloads, childcare, and household chores [34]. Sedentary lifestyle in women has been linked to a high prevalence of BMI values in the overweight/obesity range [35]. Lack of physical activity and obesity increases the vulnerability of women to NCDs [36]. Therefore, behavioral change strategies are needed to sensitize female teachers in order that they engage in sufficient physical activity. Multi-sectoral approaches are required to prioritize the establishment of open spaces and exercise facilities for women. Given the contextual constraints, Afghan women need to be sensitized through different communication channels such as mass media (e.g., television and radio) and religious sermons to engage in physical activity both inside and outside the houses. Health professionals are encouraged to provide physical activity advice during health checkups and persuade women to start or maintain habitual physical activity.

On average, consumption of fruits and vegetables more than four times per week was satisfactory among the participants; with a higher prevalence among females than males. A similar gender difference was obtained in studies conducted in Finland and Baltic countries [37], the UK [38], and some low- and middle-income countries [39]. Men and women have different levels of knowledge about healthy foods, food choices, and weight reduction. In addition, men tend to eat meals not prepared at home more frequently [40], and such meals often contain only small quantities of fruits or vegetables. Fruit and vegetable consumption is beneficial to one’s health and is recommended to reduce the risk of NCDs.

Tobacco use is a severe public health problem and a significant risk factor for NCDs. In this study, the overall prevalence of tobacco use was just over 5%, which is lower than the prevalence reported among teachers in Spain (29.7%) [41], Bangladesh (17%) [42], India (14.5%) [43], and Malaysia (7.8%) [44]. Tobacco use carries a high stigma among teachers in Afghanistan, which may be a reason for its lower prevalence. The current study also showed that tobacco use was significantly higher among males than among females. Studies conducted in India [45], Iran [46], and among schoolteachers in Turkey [47] also reported a higher prevalence of tobacco smoking among men than women. This higher smoking prevalence among men could be because tobacco use by women is considered an unfeminine trait not culturally or socially acceptable in Afghanistan. Male and female smoking trends can be linked to several social and economic factors, including gender equality, spread of smoking, economic growth, and public policies [48]. Considering the adverse health effects of tobacco use and that tobacco use by teachers can also adversely influence the attitudes of students, it is crucial to raise awareness about tobacco use hazards among teachers.

Assessment of lifestyle behaviors requires a more rigorous approach that includes a thorough analysis of all aspects of physical exercise and dietary intake relevant to NCD prevention and control. In our study, we assessed the consumption of fruits and vegetables in males and females. However, the use of detailed food frequency questionnaires will provide a valid assessment of food groups, nutrient intakes, and dietary patterns, which is essential in understanding NCD prevalence. The self-reported physical activity assessment consisted of frequency, duration, and types of daily activities. Measuring other dimensions of physical activity, such as intensity and setting wherein the physical activity is performed, is also important. Self-reported dietary habits and physical activity measurements are considerably complicated and remain a challenge in health research [49].

### 4.4. Health Professional Advice on Lifestyle Modification

Fewer than half of all the teachers reported receiving advice from health professionals on the importance of adequate fruit and vegetable intake, reduced fat consumption and reduced salt intake, habitual physical activity, weight loss, and smoking cessation. This frequency was even lower for teachers who had not visited healthcare facilities in the previous 12 months. In Afghanistan, because of the daily patient load, physicians and health professionals see a lot of patients within a limited period and they lack time to provide advice on healthy lifestyle behaviors. Advice from health professionals is an effective way of encouraging health-promoting behaviors [50]. A study of British adults showed a positive association between health professional advice to lose weight and desire and actual attempt to lose weight [51]. Health professionals are encouraged to routinely speak to health service users about lifestyle modifications at each appointment. They can also persuade people to change their practices through indirect means, including mass media [52] and distribution of health-related products to prevent and manage NCDs.

## 5. Strength and Limitations

The major strength of the study included consideration of both self-reported health behaviors and actual measured values of biomedical indicators of NCDs for analysis, which could be considered to provide a valid health profile of schoolteachers in Kabul. We measured the serum levels of HbA1c, which is useful to assess glycemic trends over a period of several months, so that the participants were not required to fast for several hours. The study sample was selected from a relatively homogeneous group— teachers—to minimize variations in the study participants’ education and income levels. Therefore, the current study results should be generalized with caution. However, the sample size was large, and the participants were selected from all 22 municipal districts of Kabul and included citizens from various provinces and ethnic groups. Other limitations should also be taken into consideration. First, fruit/vegetable consumption was assessed with a simple instrument that consisted of a few questions about food items and their frequency. Despite its reasonable reliability and validity, it was considered a weak tool to provide an accurate assessment of food groups, daily servings of fruit/vegetable consumed, nutrient intakes, and dietary patterns. Second, self-reported fruit/vegetable consumption may be subject to a social desirability bias. And finally, the questionnaire on physical activity consisted of questions on types, frequency, and duration of physical exercise/walking performed in the previous seven days. Such self-reported instruments may not be capable of harvesting more data on intensity, setting, and other domains of physical activity.

## 6. Conclusions

The present study revealed high prevalence of unhealthy lifestyle behaviors and abnormal levels of NCDs’ biomedical indicators among both male and female teachers in Afghanistan. A high prevalence of BMI values in the overweight/obesity range, increased LDL cholesterol, and physical inactivity were identified as areas of concern in female teachers. Among male teachers, the high prevalence of hypertension, increased triglycerides, habitual tobacco use, and inadequate consumption of fruits/vegetables were identified as important risk factors that need to be addressed. Improvement in access to and quality of health services and routine health screening, particularly for female teachers, should be considered to better manage NCDs in Afghanistan. Attention should be paid to encouraging teachers, particularly females, to practice healthy lifestyle behaviors, including regular physical exercise and healthy dietary habits through implementation of behavior change programs. The MoPH and sector ministries should prioritize creating a supportive environment and adopting effective policies reflecting gender differentials to improve quality of care and curb the growing burden of NCDs within the country.

## Figures and Tables

**Table 1:** Characteristics of participants stratified by gender among schoolteachers in Afghanistan (*n* = 600).

Variables	*n* (%)	% Male (*n* = 184; 30.7%)	% Female (*n* = 416; 69.3%)	*p*-Value
Age (years)				<0.001
18–40	290 (48.3)	51.6	46.9	
41–50	191 (31.8)	20.1	37.0	
≥51	119 (19.9)	28.3	16.1	
Marital status				<0.001
Never married	122 (20.3)	4.9	27.2	
Currently married	478 (79.7)	95.1	72.8	
Education level				0.026
High-school graduate	32 (5.3)	8.7	3.9	
2-year college (14th grade) graduate	362 (60.3)	54.9	62.7	
College/university graduate or higher	206 (34.4)	36.4	33.4	
Teaching experience (years)				0.119
<10	191 (31.8)	30.4	32.4	
10–20	197 (32.8)	38.6	30.3	
≥21	212 (35.4)	31.0	37.3	
Monthly income, Afghanis ^a^				<0.001
≤10,000	190 (31.7)	46.7	25.0	
10,001–20,000	272 (45.3)	41.3	47.1	
>20,001	138 (23.0)	12.0	27.9	
Self-perception of the general health status				0.003
Excellent	52 (8.7)	7.6	9.1	
Good	306 (51.0)	61.4	46.4	
Fair or poor	242 (40.3)	31.0	44.5	
Health service use in the previous 12 months				0.060
No	240 (40.0)	45.7	37.5	
Yes	360 (60.0)	54.3	62.5	

Note. ^a^ 1 USD = 66.67 Afghanis, as of January 2017.

**Table 2:** Prevalence of biomedical indicators related to non-communicable diseases, comorbidities, lifestyle behaviors, self-health check practices, and receipt of health professional advice by gender among schoolteachers in Afghanistan (*n* = 600).

Variables	Male (*n* = 184) *n* (%)	Female (*n* = 416) *n* (%)	*p*-Value
Biomedical indicators of NCDs			
High blood pressure (≥130/85 mm Hg) ^a^	50 (28.2)	79 (20.4)	0.038
High HbA1c (≥5.5%) ^b^	48 (26.4)	115 (28.8)	0.542
High cholesterol (≥200 mg/dL) ^c^	29 (15.8)	80 (20.5)	0.185
High LDL cholesterol (≥100 mg/dL) ^c^	93 (50.8)	239 (61.3)	0.018
Low HDL cholesterol (<40 mg/dL) ^c^	44 (24.0)	116 (29.7)	0.156
High triglyceride level (≥150 mg/dL) ^c^	86 (47.0)	148 (37.9)	0.040
Overweight/obesity (BMI≥25.0 kg/m^2^)	80 (43.5)	269 (64.7)	<0.001
Multiple unfavorable conditions			
Three or more ^a,b,c^	71 (40.8)	168 (46.1)	0.243
Lifestyle behaviors			
Physical exercise or walking (≥1 h per day)	100 (54.3)	170 (40.9)	0.002
Consumption of fruits/vegetables (≥4 times per week)	99 (53.8)	297 (71.4)	<0.001
Tobacco use	29 (15.8)	3 (0.7)	<0.001
Self-health check practices			
Measured blood pressure	76 (41.3)	218 (52.4)	0.012
Measured blood glucose	31 (16.8)	126 (30.3)	0.001
Measured cholesterol	30 (16.3)	126 (30.3)	<0.001
Health professional advice on lifestyle modifications			
Eat at least five servings of fruits/vegetables each day	82 (44.6)	215 (51.7)	0.108
Reduce fat intake	74 (40.2)	222 (53.4)	0.003
Reduce salt intake	61 (33.1)	177 (42.5)	0.030
Start physical exercise or increase physical activities	78 (42.4)	216 (51.9)	0.031
Lose/maintain body weight	62 (33.7)	193 (46.4)	0.004
Avoid tobacco use	30 (16.3)	68 (16.3)	0.990

Note. HbA1c, glycosylated hemoglobin; LDL, Low-density lipoprotein; HDL, High-density lipoprotein; BMI, Body mass index. ^a^ Excluded teachers who were on medication for hypertension (*n* = 35). ^b^ Excluded teachers who were on medication for diabetes (*n* = 19). ^c^ Excluded teachers who were on medication for hyperlipidemia (*n* = 27).

**Table 3:** Distribution of mean levels of biomedical indicators related to non-communicable diseases by age and gender among schoolteachers in Afghanistan (*n* = 600).

Variables	Age	Mean (95% CI)		
		Male (*n* = 184)	Female (*n* = 416)	Total (*n* = 600)
Systolic blood pressure (mm Hg) ^a^	18–40	117.3 (115.5–119.1)	113.7 (112.4–115.0)	114.9 (113.8–116.0)
	41–50	124.7 (119.9–129.6)	122.5 (120.5–124.6)	123.0 (121.1–124.9)
	≥51	128.0 (124.2–131.8)	122.6 (119.5–125.8)	125.1 (122.6–127.6)
Diastolic blood pressure (mm Hg) ^a^	18–40	77.9 (76.5–79.4)	74.6 (73.6–75.7)	75.7 (74.8–76.6)
	41–50	82.4 (79.0–85.8)	80.7 (79.3–82.2)	81.1 (79.7–82.4)
	≥51	84.3 (81.6–87.0)	80.9 (78.5–83.2)	82.4 (80.7–84.2)
HbA1c (%) ^b^	18–40	5.3 (5.0–5.5)	5.1 (5.0–5.2)	5.2 (5.1–5.2)
	41–50	5.5 (5.2–5.7)	5.6 (5.4–5.7)	5.5 (5.4–5.7)
	≥51	5.6 (5.3–5.9)	5.8 (5.4–6.1)	5.7 (5.5–5.9)
Cholesterol (mg/dL) ^c^	18–40	157.7 (151.1–164.3)	162.8 (158.2–167.3)	161.1 (157.3–164.9)
	41–50	171.5 (162.2–180.8)	180.2 (174.0–186.4)	178.4 (173.1–183.7)
	≥51	173.2 (161.2–185.2)	185.2 (176.2–194.2)	179.7 (172.3–187.1)
LDL cholesterol (mg/dL) ^c^	18–40	98.4 (92.4–104.4)	102.7 (98.8–106.7)	101.3 (98.0–104.6)
	41–50	111.4 (102.5–120.3)	116.7 (111.6–121.8)	115.6 (111.2–120.0)
	≥51	110.8 (101.4–120.2)	123.5 (115.8–131.2)	117.6 (111.6–123.7)
HDL cholesterol (mg/dL) ^c^	18–40	42.1 (41.3–42.9)	41.7 (41.2–42.2)	41.8 (41.4–42.8)
	41–50	41.1 (40.1–42.1)	39.6 (38.8–40.4)	39.9 (39.3–40.6)
	≥51	40.2 (38.9–41.6)	39.0 (37.9–40.0)	39.5 (38.7–40.4)
Triglycerides (mg/dL) ^c^	18–40	158.2 (143.4–173.1)	136.8 (127.4–146.3)	143.9 (135.8–152.0)
	41–50	194.9 (161.1–228.8)	160.1 (148.7–171.5)	167.5 (155.8–179.1)
	≥51	171.2 (141.0–201.3)	164.4 (140.5–188.3)	167.5 (148.6–186.4)
BMI (kg/m^2^)	18–40	23.8 (23.0–24.5)	25.9 (25.2–26.5)	25.2 (24.6–25.7)
	41–50	25.8 (24.4–27.2)	28.4 (27.6–29.2)	27.9 (27.2–28.6)
	≥51	24.9 (23.9–25.9)	27.1 (26.1–28.1)	26.1 (25.4–26.9)

Note. HbA1c, glycosylated hemoglobin; LDL, Low-density lipoprotein; HDL, High-density lipoprotein; BMI, Body mass index; CI, Confidence interval. ^a^ Excluded teachers who were on medication for hypertension (*n* = 35). ^b^ Excluded teachers who were on medication for diabetes (*n* = 19). ^c^ Excluded teachers who were on medication for hyperlipidemia (*n* = 27).

**Table 4:** Self-health check practices, receipt of health professional advice, and lifestyle behaviors by gender and status of health service use in the previous 12 months among schoolteachers in Afghanistan (*n* = 600).

Variables	Overall		*p*-Value	Male		*p*-Value	Female		*p*-Value
	% Users (*n* = 360)	% Non-Users (*n* = 240)		% Users (*n* = 100)	% Non-Users (*n* = 84)		% Users (*n* = 260)	% Non-Users (*n* = 156)	
Self-health check practices									
Measured blood pressure	56.1	38.3	<0.001	49.0	32.1	0.021	58.8	41.7	0.001
Measured blood glucose	32.8	16.2	<0.001	19.0	14.3	0.395	38.1	17.3	<0.001
Measured cholesterol	32.2	16.7	<0.001	19.0	13.1	0.280	37.3	18.6	<0.001
Health professional advice on lifestyle modifications									
Eat at least five servings of fruits/vegetables each day	55.3	40.8	0.001	46.0	42.9	0.669	58.8	39.7	<0.001
Reduce fat intake	55.6	40.0	<0.001	43.0	36.9	0.401	60.4	41.7	<0.001
Reduce salt intake	43.9	33.3	0.010	34.0	32.1	0.790	47.7	34.0	0.006
Start physical exercise or increase physical activities	55.0	40.0	<0.001	48.0	35.7	0.093	57.7	42.3	0.002
Lose/maintain body weight	47.8	34.6	0.001	39.0	27.4	0.097	51.1	38.5	0.012
Avoid tobacco use	15.6	17.5	0.528	14.0	19.0	0.356	16.1	16.7	0.891
Lifestyle behaviors									
Physical exercise or walking (≥1 h per day)	41.4	50.4	0.029	53.0	55.9	0.689	36.9	47.4	0.035
Consumption of fruits/vegetables (≥4 times per week)	64.4	68.3	0.325	52.0	55.9	0.592	69.2	75.0	0.207
Tobacco use	4.4	6.7	0.235	13.0	19.0	0.262	1.1	0.0	0.178

**Table 5:** Biomedical indicators related to non-communicable diseases and comorbidities by gender and status of health service use in the previous 12 months among schoolteachers in Afghanistan (*n* = 600).

Variables	Overall		*p*-Value	Male		*p*-Value	Female		*p*-Value
	% Users (*n* = 360)	% Non-Users (*n* = 240)		% Users (*n* = 100)	% Non-Users (*n* = 84)		% Users (*n* = 260)	% Non-Users (*n* = 156)	
Biomedical indicators of NCDs									
High blood pressure (≥130/85 mm Hg) ^a^	21.9	24.1	0.537	33.0	22.5	0.123	17.4	25.0	0.069
High HbA1c (≥5.5%) ^b^	27.5	28.9	0.715	28.6	23.8	0.467	27.0	31.6	0.327
High cholesterol (≥200 mg/dL) ^c^	21.5	15.4	0.071	20.0	10.8	0.091	22.1	18.0	0.331
High LDL cholesterol (≥100 mg/dL) ^c^	59.4	55.8	0.389	58.0	42.2	0.033	60.0	63.3	0.511
Low HDL cholesterol (<40 mg/dL) ^c^	30.0	24.5	0.126	29.0	18.1	0.085	30.8	28.0	0.552
High Triglycerides (≥150 mg/dL) ^c^	40.9	40.8	0.979	49.0	44.6	0.551	37.5	38.7	0.817
Overweight/obesity (BMI ≥ 25.0 kg/m^2^)	60.3	55.0	0.199	48.0	38.1	0.177	65.0	64.1	0.853
Multiple unfavorable conditions									
Three or more ^a,b,c^	44.9	43.8	0.806	45.3	35.4	0.189	44.7	48.3	0.499

Note. HbA1c, glycosylated hemoglobin; LDL, Low-density lipoprotein; HDL, High-density lipoprotein; BMI, Body mass index. ^a^ Excluded teachers who were on medication for hypertension (*n* = 35). ^b^ Excluded teachers who were on medication for diabetes (*n* = 19). ^c^ Excluded teachers who were on medication for hyperlipidemia (*n* = 27).

## Data Availability

The data presented in this study are available on request from the corresponding author. The data are not publicly available due to participants’ confidentiality.

## Supplementary Materials

The following are available online at https://www.mdpi.com/article/10.3390/ijerph18115729/s1, Table S1. Prevalence of lifestyle behaviors by biomedical indicators related non-communicable diseases stratified by gender among schoolteachers in Afghanistan (*n* = 600).
