# Supplementary material for: Gender-Specific Prevalence of Risk Factors for Non-Communicable Diseases by Health Service Use among Schoolteachers in Afghanistan
Source: Int J Environ Res Public Health. 2021 May 26;18(11):5729. doi: 10.3390/ijerph18115729 (PMC8198773; doi:10.3390/ijerph18115729)
Supplement: Supplementary file 1 [file ijerph-18-05729-s001.zip › ijerph-1198364-supplementary.pdf]

International Journal of
Environmental Research and Public Health

MDPI

Table S1. Prevalence of lifestyle behaviors by biomedical indicators related non-communicable diseases stratified by gender among schoolteachers in Afghanistan (n = 600).

**Lifestyle Behaviors**	**High Blood Pressure (≥130/85 mm Hg)[a]**	**High HbA1c (≥5.5%)[b]**	**High Cholesterol (≥200 mg/dL)[c]**	**High LDL Cholesterol (≥100 mg/dL)[c]**	**Low HDL Cholesterol (<40 mg/dL)[c]**	**High Triglyceride Level (≥150 mg/dL)[c]**	**Overweight/Obesity (BMI ≥ 25.0 kg/m²)**
				Male			
Physical exercise or walking							
<1 h per day	24 (30.0)	23 (28.1)	13 (15.5)	49 (58.3)	18 (21.4)	42 (50.0)	41 (48.8)
≥1 h per day	26 (26.8)	25 (25.0)	16 (16.2)	44 (44.4)	26 (26.3)	44 (44.4)	39 (39.0)
p-Value	0.638	0.642	0.899	0.061	0.446	0.453	0.181
Consumption of fruits/vegetables							
<4 times per week	26 (32.5)	19 (22.3)	16 (18.8)	45 (52.9)	23 (27.1)	40 (47.1)	40 (47.1)
≥4 times per week	24 (24.7)	29 (29.9)	13 (13.3)	48 (49.0)	21 (21.4)	46 (46.9)	40 (40.4)
p-Value	0.254	0.249	0.304	0.593	0.374	0.987	0.364
Tobacco use							
No	40 (26.8)	39 (25.3)	21 (13.6)	75 (48.7)	75 (48.7)	69 (44.8)	67 (43.2)
Yes	10 (35.7)	9 (32.1)	8 (27.6)	18 (62.1)	18 (62.1)	17 (58.6)	13 (44.8)
p-Value	0.339	0.451	0.059	0.187	0.187	0.171	0.873
				Female			
Physical exercise or walking							
<1 h per day	50 (21.8)	69 (29.7)	48 (20.6)	141 (60.5)	67 (28.8)	91 (39.1)	157 (63.8)
≥1 h per day	29 (18.2)	46 (27.5)	32 (20.4)	98 (62.4)	49 (31.2)	57 (36.3)	112 (65.9)
p-Value	0.387	0.633	0.958	0.705	0.603	0.583	0.665
Consumption of fruits/vegetables							
<4 times per week	24 (21.2)	29 (25.2)	25 (21.9)	73 (64.0)	38 (33.3)	49 (43.0)	71 (59.7)

≥4 times per week	55 (20.0)	86 (30.3)	55 (19.9)	166 (60.1)	78 (28.3)	99 (35.9)	198 (66.7)
p-Value	0.783	0.312	0.656	0.473	0.319	0.188	0.177
Tobacco use							
No	79 (20.5)	114 (28.7)	80 (20.7)	237 (61.2)	237 (61.2)	147 (38.0)	268 (64.9)
Yes	0 (0.0)	1 (50.0)	0 (0.0)	2 (66.7)	2 (66.7)	1 (33.3)	1 (33.3)
p-Value	0.473	0.507	0.377	0.848	0.848	0.869	0.255

Note. HbA1c, glycosylated hemoglobin; LDL, Low-density lipoprotein; HDL, High-density lipoprotein; BMI, Body mass index. [a] Excluded teachers who were on medication for hypertension ($n = 35$). [b] Excluded teachers who were on medication for diabetes ($n = 19$). [c] Excluded teachers who were on medication for hyperlipidemia ($n = 27$).
